# Identification association of drug-disease by using functional gene module for breast cancer

**DOI:** 10.1186/1755-8794-8-S2-S3

**Published:** 2015-05-29

**Authors:** Lida Zhu, Fuxi Zhu

**Affiliations:** 1 Agricultural bioinformatics Key Laboratory of HuBei Province, College of Informatics, Huazhong Agricultural University Wuhan, P.R. China; 2 School of Computer, Wuhan University Wuhan, P.R. China

**Keywords:** gene module, prognostic associated, drug sensitivity module, breast cancer

## Abstract

In oncology drug development, it is important to develop low risk drugs efficiently. Meanwhile, computational methods have been paid more and more attention in drug discovery. However, few studies attempt to discover the mutual gene modules shared by the drug and disease association. Here we introduce a novel method to identify repositioned drug for breast cancer by integrating the breast cancer survival data with the drug sensitivity information. Among the 140 drug candidates, we are able to filter 4 FDA approved drugs and identify 2 breast cancer drugs among 4 known breast cancer therapeutic drug in total.

## Introduction

The goal of drug repositioning is to discover new association between indications of diseases and known drugs based on known associations [1]. Drug repositioning provides a possible way to speed up the drug development and avoids the development cost and time consuming in the drug discovery. For example, the Pfizer's Viagra and Celgene's thalidomide are both successful examples that are used to treat new application rather than the designed indication during the development stage [2-4]. However, the mechanism of how drugs and diseases are associated in the gene regulatory network is still mysterious. Therefore, the need of developing a systematic approach to discover disease-drug association from the complicate heterogeneity data is urgent.

Roughly speaking, both human diseases and drug mode of action have a module basis on gene expression level. The rapidly developed microarray technique generates a huge amount of large-scale gene expression profiles. The gene expression data are both applied in cancer diagnosis and therapy. To identify the phenotype relevant gene modules could help us understand the mechanism of diseases and provides opportunities to develop new therapies. It is important to investigate the drug induced gene modules, and the differential expressed functional gene modules that influenced by diseases, especially the shared associated gene modules of drugs and disease phenotypes. Few analyses addressed the above questions in a systematic view. Gottlieb et al. presented a prediction method that based on the observation that similar drugs are indicated for similar diseases, and then discovers new associations by imposing the known "drug-disease" connections. Cheng et al. proposed a network-based inference method to predict new target for known drugs by using drug target bipartite network topology similarity [5,6]. With the emerging development of the microarray technology, some researches focused on using the gene expression data as the connection between drug and disease for drug repositioning. For example, "the Connectivity Map" (CMAP) project developed a reference collection of gene expression profiles by treating cultured human cells with small molecules, and searched for the compounds that have the most negative correlation with the disease's gene expression profiles as the potential candidate [7,8]. However, due to the limitation of the level of gene expression profiles among the above works, the molecular mechanism of diseases still remains unknown. Recently, some studies suppose that cellular components belonging to the same topological, functional or disease module have a high likelihood of being involved in similar diseases [9,10]. Thus one can start with identifying the gene modules that associate with the topological, functional and disease. The gene modules here is defined by the group of genes that annotated by biological process or cellular component or molecular function terms in Gene Ontology [11,12]. There are several studies proposed to characterize different disease modules. The investigation on drug action of the gene modules had shown effective results. Li et al., by using Gene Ontology, defined the Gene Ontology Module (GOM) and investigated the disease's biological mechanism, and then successfully identified several repositioned drugs by using the similarity of GOM [13]. Xia et al. used Gene Ontology Term to define the miRNA target gene's function in various disease, and used it to predict several potential treatments [14]. Since the prognosis studies were also sampled by high throughput gene expression profiles, in this work we present how to make use of this kind of clinical prognosis data and initiate the mutual gene modules to investigate drug repositioning for the treatment of breast cancer. The definition of the mutual gene module of disease is based on the Gene Ontology Cellular Component Terms and Gene Ontology Biological Process Term. We selected the gene modules that have significant association with tumor progression by applying t-test in multiple prognostic clinical trials. In the meanwhile, we define a sensitivity module of drugs to interrogate the mechanism of drug actions. The sensitivity module for each anti-cancer drug (in total 140) is generated by integrating the sensitivity data (e.g. GI_50_) from the Developmental Therapeutic Program (DTP http://dtp.nci.nih.gov/) database. Combining with the prognosis modules and drug sensitivity modules, a repositioning framework for breast cancer is built. As a result, we have identified 12 compounds related to breast cancer. Interestingly, Not only could they help in treatment, but some of them could also have caused the disease.

## Material and Method

**Figure 1:**
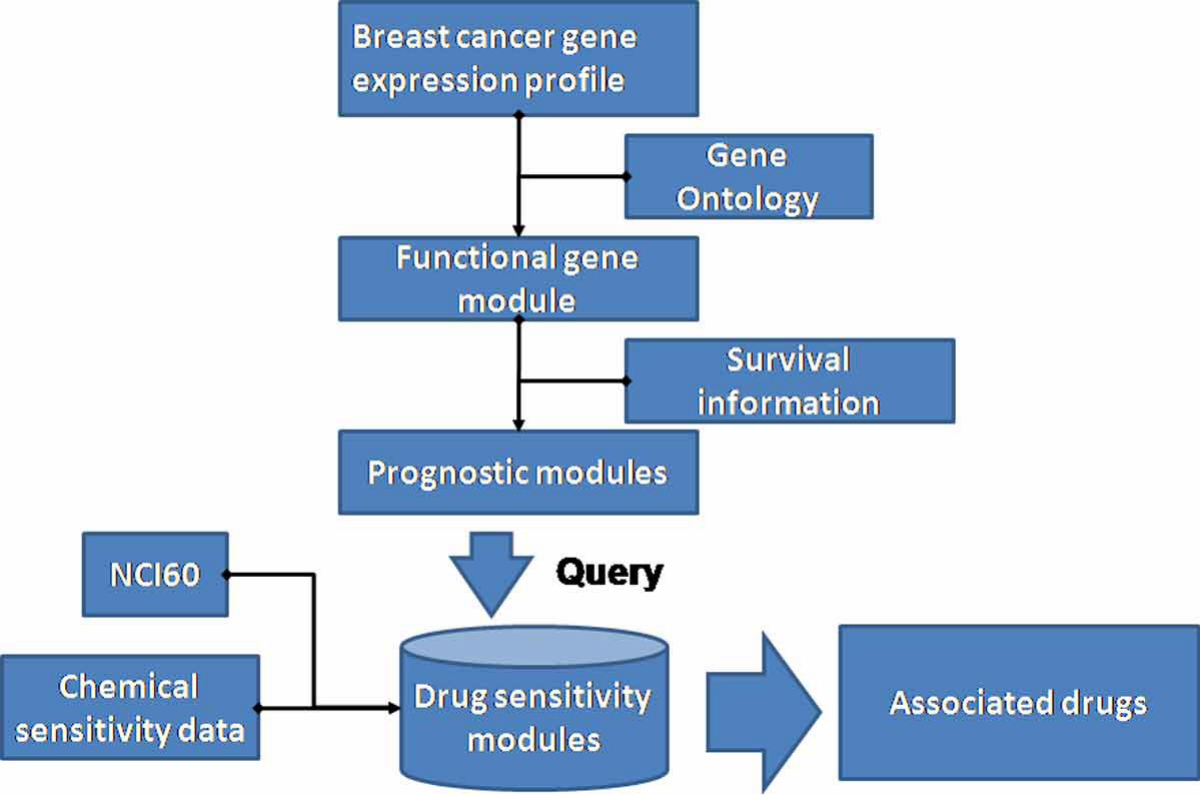
**The workflow of the method that integrated the prognostic gene modules with the drug sensitivity modules**.

Here we proposed a systematic framework to identify the potential candidate drug for breast cancer and the associated mechanism module. The framework consists of three parts: (1) Identifying the prognosis module. The prognosis module is selected based on the statistical differences between the good outcome and bad outcome of the patients in three breast cancer clinical trials. (2) Generation of the sensitivity module network of drugs. The sensitivity module network of drugs is used to interrogate the mechanism of drug action. (3) Integration of the two results to search for the potential candidate drug. In the last step, we integrated the disease gene modules with the anti-cancer drug sensitivity module to filter the most significant potential anticancer treatment for breast cancer as shown in Figure 1.

### Data description

The tumor progression associated information is collected from three breast cancer datasets (GSE2034 [15], GSE7390 [16] and GSE11121 [17]). These data are used to select the differential expressed gene modules associated to the prognosis outcome among all the patient samples.

The drug information was downloaded from DTP database, which contains 172 anti-cancer compounds in total [18,19]. The format of the analyzed data is shown in Table 1. After matched with the GI_50 _value in 60 NCI cell lines [20], the sensitivity geneset was generated for the remaining140 drugs.

**Table 1:** The format of the analyzed drug.

NSC	LCONC	PANEL	CELL	NLOGGI50	Compound	CAS
740	-5	Renal	786-0	7.644	methotrexate	1959-5-2
740	-5	Renal	A498	5	methotrexate	1959-5-2
740	-8.6	Non-Small Cell Lung	A549-ATCC	8.602	methotrexate	1959-5-2
740	-3.6	Renal	ACHN	7.308	methotrexate	1959-5-2
740	-3.6	Breast	BT-549	4.651	methotrexate	1959-5-2
740	-3.6	Renal	CAKI-1	7.124	methotrexate	1959-5-2
740	-3.6	Leukemia	CCRF-CEM	7.508	methotrexate	1959-5-2
740	-8.6	Colon	COLO 205	8.602	methotrexate	1959-5-2
740	-5	Prostate	DU-145	7.238	methotrexate	1959-5-2
740	-8.6	Non-Small Cell Lung	EKVX	8.602	methotrexate	1959-5-2
740	-5	Colon	HCC-2998	7.025	methotrexate	1959-5-2
740	-8.6	Colon	HCT-116	8.89	methotrexate	1959-5-2

### Gene module enrichment analysis

A gene module was defined by a group of genes that sharing a similar function or regulation mechanism. Here we used the Gene Ontology as an example. The Gene Ontology was annotated into three categories: Biological Process (GOBP), Cellular Component (GOCC) and Molecular Function (GOMF). The annotated Gene Ontology Term was considered as a gene module.

To determine the universal prognosis gene modules that represent breast cancer pathologic information, gene set enrichment analysis is performed on three different breast cancer survival datasets, with the purpose of selecting the gene sets that express significantly enriched in the survived patients. First, the gene sets were adopted from GOCC and GOBP. For each breast cancer expression dataset, we have *n *samples. Then the Kolmogorov-Smirnov (KS) statistic is employed to test whether the expression of the gene set is enriched among the whole gene expression (N) in each sample. The KS is calculated as following formulas: [13]

(1) $$a=\underset{j}{Max}\;t\left[\frac{j}{t}-\frac{V\left(j\right)}{N}\right]$$

(2) $$b=\underset{j}{Max}\;t\left[\frac{V\left(j\right)}{N}-\frac{j-1}{t}\right]$$

(3) $$KS_{}=\left\{\begin{array}{c} -b,\;\;a<b\\ \;a,\;\;a>b \end{array}\right)$$

where *t *is the number of the genes in the gene set, *j *is the *j^th ^*gene according to the ranked expression in the gene set, N is the total number (13698) in the gene expression profile, *V(j) *is the ranked position of the *j^th ^*gene, and KS score represents the enrichment score of each gene set in each sample. The result of this analysis is a matrix generated by the enrichment score of each gene module in each sample.

### Identification of prognosis gene modules

For each disease dataset, we used the t-test (as shown in formula (4)) to find whether the gene set has significant differential enrichment scores between the good outcome and bad outcome. And then we selected those gene sets with significant P-value (<0.05) among three clinical trials.

(4) $$t=\frac{\bar{x}-\bar{y}}{\sqrt{\frac{s_{x}^{2}}{n}+\frac{s_{y}^{2}}{m}}}$$

where *x *stands for the average enrichment score of the survived patient, *y *stands that of the patient with bad outcome, *n *is the number of the survived patients, and *m *is the number of the rest of the patients.

### Identification of drug sensitivity genes

Here we measure the drug sensitivity signature based on National Cancer Institute (NCI60) *in vitro *drug screen project [20]. The subset of compounds in this study was defined in the "Standard Agent Database" from DTP website [18,19]. The drug sensitivity genes were identified by the close correlation of the baseline expression of gene among 60 cell lines with the chemical activity of a treated drug on the same 60 cell lines. On one hand, since the NCI60 cell lines screen panel has proved to be an effective way to identify drug sensitivity specific biomarkers as the panel has already been comprehensively characterized via gene expression profiling. Therefore, the gene expression profile of NCI60 is used to measure the baseline of gene expression and represented by a matrix of gene expression profile in 60 cell lines. On the other hand, the biological response pattern is measured by GI_50 _value, which is the compound's concentration that causes 50% cell growth inhibition and was obtained from DTP. Since the GI_50 _value is represented the drug's chemo-activity, the gene-drug correlation is established based on the correlation of gene expression and drug activity across 60 cell lines. The sensitivity module is consisted of only the significant gene that has P-value<0.05 as the compound sensitivity associated gene.

### Generalization of the drug-disease connection

The drug-disease link is established based on the P-value of the hyper-geometric test (5) to determine whether the gene module (from the selected prognosis gene modules) has a significant overlap with the sensitivity module of the drug.

(5) $$P=\underset{x\geq n}{\sum }\frac{C_{N}^{x}\cdot C_{M-N}^{m-x}}{C_{M}^{m}}$$

where *M *is the total number of genes in the whole profiles, *N *is the number of the prognosis gene modules, *m *is the number of the sensitivity module of the drug, and *n *is the number of the overlap between the prognosis module and the sensitivity module.

### The performance analysis of the identification of drugs

As shown in the final step of Figure 1, a ranked list of the modules and the selected prognosis modules was generated with the most significant connection to the prognosis.

The performance of the drug identification is assessed by the Effect Score which is the number of prognosis gene modules with significance divided by the half number of the identified prognosis gene modules. According to the ES, we estimated our performance by counting on the number of positive drug in the ranked drug list.

## Result

### Characterization of drug sensitivity genes

To assess the potential application of this approach for identifying compounds, we generated the sensitivity module for each anti-cancer drug by applying Pearson correlation coefficient on the GI_50 _value of each drug with the NCI60 profiling. After this process, we gathered 140 compounds sensitivity gene sets. The genes which have significance connection between the biological response pattern (GI_50_) and the baseline gene expression, though may not be directly linked to the primary drug targets, they should be close to the pathway where the mechanism of action taking place. Thus these genes can indicate the key biological processes of drug efficacy and represent the sensitivity genes of a compound which can serve as an effective tool for probing the compounds mechanism of action.

### Identification of prognosis gene modules of breast cancer

In order to identify the tumor progression associated gene modules, we collected three datasets of breast cancer, GSE2034 [15], GSE7390 [16] and GSE11121 [17]. Based on the enrichment score of the gene modules in each dataset, we used t-test to select the gene modules that has significant statistical different enrichment score across the patient samples of outcome. Then we selected the overlap gene modules that shown significantly associated to prognosis in all the data. The 30 gene modules were identified and listed in Table 2. As shown in Table 2, the prognosis modules are most likely related to the biologic process of cell cycle, and microtubule related biologic processes and cellular components, which was generally reported that has close association with cancer progression [21].

**Table 2:** Identified Breast cancer Prognostic Gene Ontology Modules

	Prognostic Gene Ontology Modules
1	BP:mitotic spindle elongation
2	BP:M phase of mitotic cell cycle
3	BP:mitotic cell cycle
4	BP:M phase
5	BP:cytokinesis, actomyosin contractile ring assembly
6	BP:negative regulation of type 2 immune response
7	BP:epithelial cell morphogenesis
8	BP:DNA replication
9	BP:organelle organization
10	BP:microtubule-based movement
11	BP:cell cycle
12	BP:mitotic cell cycle
13	BP:M phase
14	BP:cytokinesis, actomyosin contractile ring assembly
15	BP:regulation of asymmetric cell division
16	BP:cell cycle process
17	BP:chromosome condensation
18	BP:protection from non-homologous end joining at telomere
19	BP:cell cycle cytokinesis
20	BP:meiotic chromosome segregation
21	BP:negative regulation of sister chromatid cohesion
22	BP:cell division
23	BP:maintenance of centrosome location
24	BP:regulation of chromosome segregation
25	BP:mitotic cell cycle G2/M transition decatenation checkpoint
26	CC:condensed nuclear chromosome
27	CC:spindle microtubule
28	CC:DNA topoisomerase complex (ATP-hydrolyzing)
29	CC:chromosome passenger complex
30	CC:macropinocytic cup

### Investigation on Disease-Drug connection

**Figure 2:**
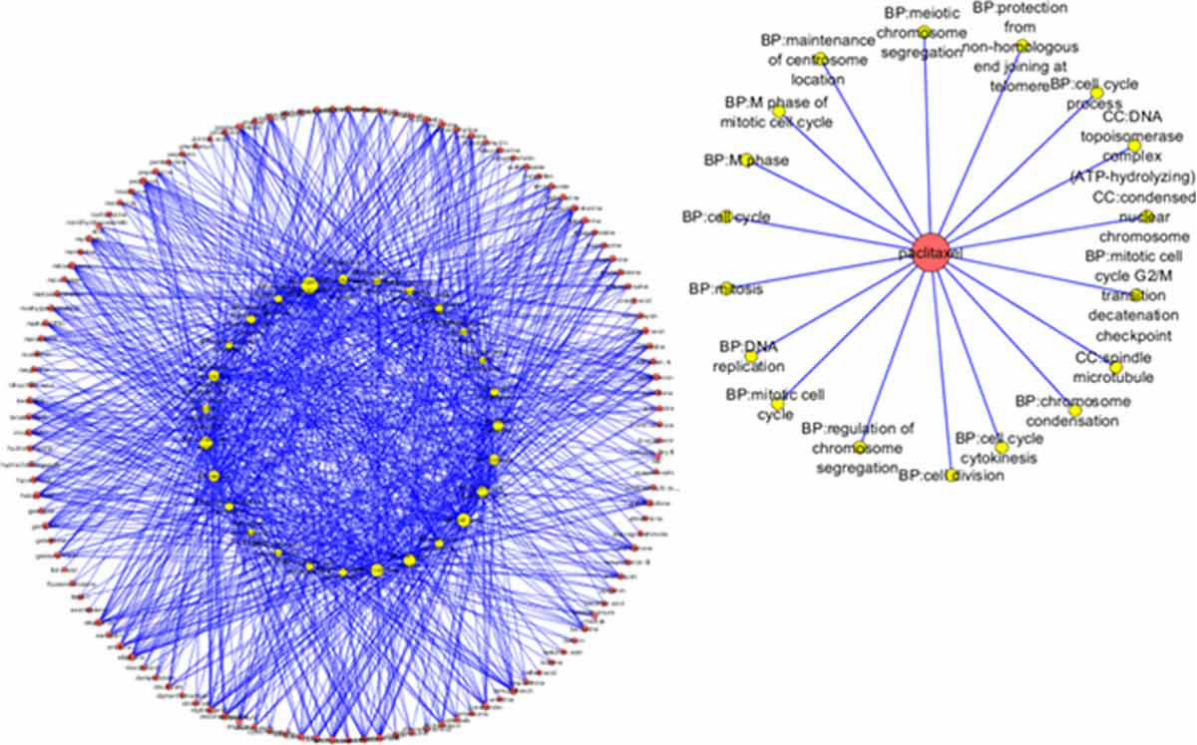
**The distribution of the Drug-Disease network**. The yellow node repersents the prognostic gene module of breast cancer. The red node is denoted for the drug sensitivity module. If the sensitivity module has a significant overlap between the prognostic gene modules, it means this module is sensitive to this drug which is denoted as a link from the red note to the yellow node. The right circle is the Paclitaxel related gene modules network.

**Figure 3:**
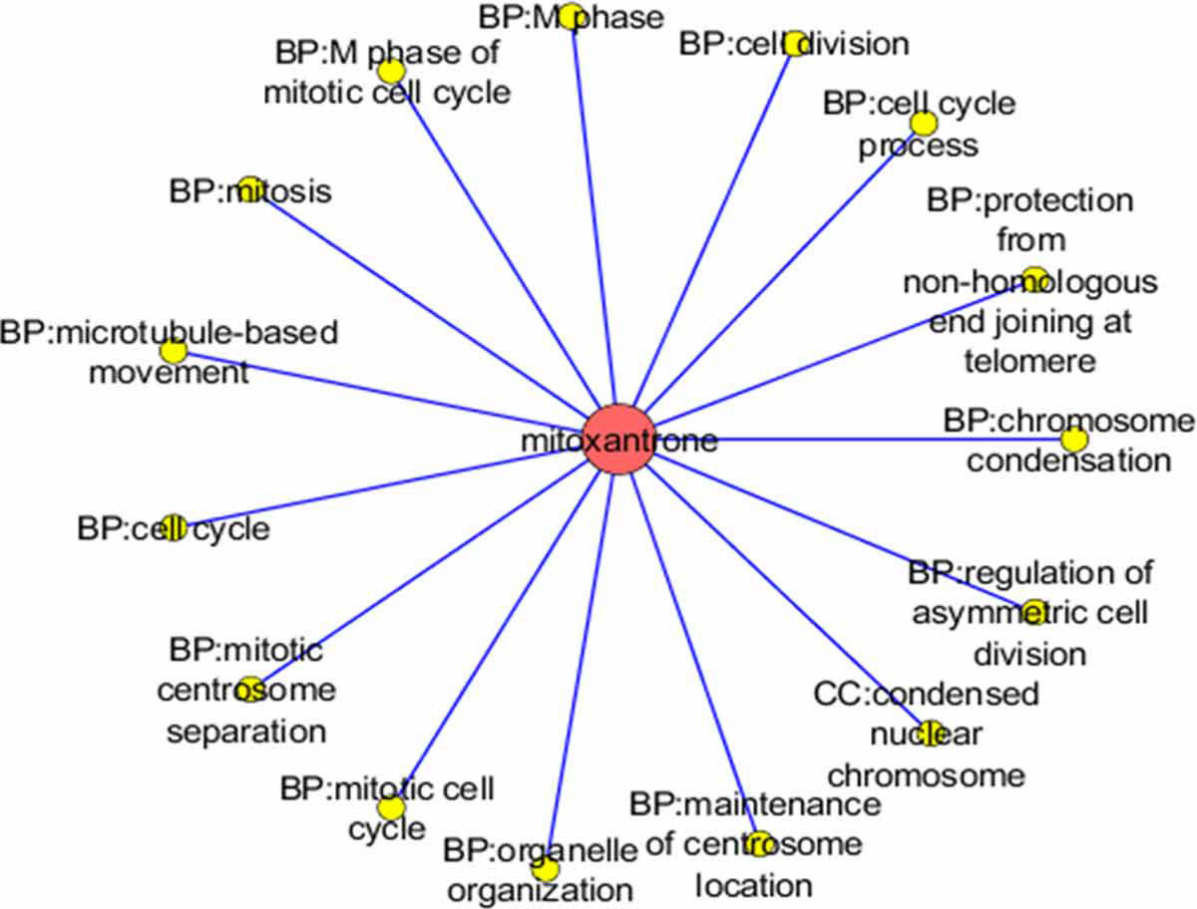
**The Mitoxantrone associated with gene modules network**.

In order to investigate whether a drug has strong effect on the gene function module, we used hyper geometric test on drug sensitivity module with the identified breast cancer prognostic associated gene module. The corresponding P-value of the hyper geometric test of each gene module was cut off by 0.05 as significance threshold. By ranking the number of the associated gene module, the FDA approved drugs such as Paclitaxel and Etoposide, Haloperidol, Mitoxantrone shows connections with more than half of the gene modules. Paclitaxel is a mitotic inhibitor used in cancer chemotherapy [22]. And it is a FDA approved drug that used to treat patients with breast and several other kinds of cancers. The Paclitaxel associated gene modules also shows related significantly with mitotic cell cycle in the biological process [23]. As shown in Figure 2 the result also shows that the microtubule of Cellular Component is sensitive to the drug action of Paclitaxel which has been validated from literature [24]. Mitoxantrone is also a FDA approved breast cancer treatment. It is a type II topoisomerase inhibitor which is an important protein for cell mitosis [25]. As listed in Table 3, it has 15 associated gene modules. Furthermore the cell mitosis related biological processes show significant sensitivity to Mitoxantrone as shown in Figure 3.

**Table 3:** Ranked list of predicted drug that related to breast cancer.

	Drug name	FDA approved	Number of associated disease gene module
1	Paclitaxel	1	18
2	Ellipticine	0	18
3	camptothecin	0	17
4	Digitoxigenin	0	17
5	tetrahydroalstonine	0	17
6	Etoposide	1	16
7	Haloperidol	1	16
8	cicloheximide	0	15
9	Genistein	0	15
10	mitoxantrone	1	15
11	podophyllotoxin	0	15
12	Securinine	0	15

Etoposide is also a topoisomerase inhibitor but haven't been reported to treat breast cancer [26]. Our result suggests that etoposide may have potential to experiment more on breast cancer treatment. Haloperidol, on the other hand, is not reported as a breast cancer treatment drug, but has pharmacological effect on increase patients' risk of breast cancer among women. As a anti-psychotics drug, it has been reported that generally links to especially women breast tumor growth [27]. This result shows that our method could recognize the compound's hidden side effect that could not be observed during the development stage.

Several not FDA approved drugs was also found has strong association with breast cancer. For example, Ellipticine was ranked also has 18 modules, and it was also reported that can act as an inhibitor to breast cancer. However, it fails for its side effect during the development [28]. Also, camptothecin, genistein also found to have strong pharmacological efficiency on breast cancer but failed by its side effect.

### Comparison of gene signatures performance

For comparison, we used gene signature instead of gene module in our method. The prognostic gene signature was extracted from GSE2034. We chose 76 genes that are reported in Wang's work [15] as the most significantly related to the prognosis risk. The gene signature was identified by the same procedure as gene module identification. Then a ranked list of drugs was produced by the number of genes overlapped with drug sensitivity modules (Table 4). Since the corresponding P values are above the threshold of 0.05. The result indicates that the gene signature has the ability to predict prognosis, however it is not very stable in the identification of useful drugs for breast cancer.

**Table 4:** Ranked list of predicted drug by method based on gene expression.

	Drug name	FDA approved	Number of associated disease gene module	Pvalue
1	Miconazole	0	33	1.93E-01
2	Monastrol	0	33	1.95E-01
3	Trifluoperazine	1	33	4.68E-02
4	Menadione	1	32	8.72E-02
5	Geldanamycin	0	31	4.40E-02
6	Cantharidin	0	29	1.64E-01
7	Ciclosporin	1	29	4.48E-02
8	Lithocholic acid	0	28	1.54E-01
9	Benzethonium chloride	0	27	1.70E-01
10	Pyrimethamine	0	27	1.86E-01
11	Tetrandrine	0	26	1.16E-01
12	Triamcinolone	1	26	1.93E-01

## Discussion and conclusion

In this study, we introduced a new pipeline to interrogate the transient cellular state under drug conditions by prognosis gene modules. Different from other methods which directly map the expression profiles of drug treatment to disease, our method was used to identify the cellular responses of drugs and gene modules that correlated with tumor progression through patients' survival information. We have identified 30 significant biological processes related to breast cancer prognosis gene modules. By integrating two results of gene modules, we provided valuable prediction information of breast cancer potential treatment. Among the drug library, we identified 12 drugs for breast cancer treatment. 4 of them are FDA approved treatment drugs, which shows the efficient of our result.

We provide a novel work to investigate on the drug sensitivity module by using the prognostic related gene module annotation and use the connection to discover new association between the drug and disease. Our work proved that the sensitivity module of drugs could be used to not only identify the drug to treat the disease, but also reveal the drug's potential side effect.

This method provides a new pipeline to identify the repositioned compound for diseases. However, there are several parts that could be improved in our future research. (1) By adopt with different cancer information, we could investigate more prognostic related gene modules. (2) The gene module could be extended according to the definitions of Protein-Protein-Interaction-Network level, or pathway information, or miRNA regulation. (3) From the disease annotated drug sensitivity module, such as the Mitoxantrone in Figure 3, it is possible to further investigation on the drug similarity. We will further improve our method and make performance comparison with other works.

## Competing interests

The authors declare that they have no competing interests.

## Authors' contributions

The work presented here was carried out in collaboration between all authors. Lida Zhu and Fuxi Zhu defined the research theme. Lida Zhu designed methods and experiments, carried out the laboratory experiments, analyzed the data, interpreted the results. Lida Zhu and Fuxi Zhu co-wrote the paper.

## Declarations

Publication of this article has been funded by the grant from the National Natural Science Foundation of China (Grant 61272277, 21173092 and 31402231), the Natural Science Foundation of Hubei Province (Grant 2013CFA016) and the Fundamental Research Funds for the Central Universities (Grant 2662014BQ081). The funders had no role in study design, data collection and analysis, decision to publish, or preparation of the manuscript.

This article has been published as part of *BMC Medical Genomics *Volume 8 Supplement 2, 2015: Selected articles from the 4th Translational Bioinformatics Conference and the 8th International Conference on Systems Biology (TBC/ISB 2014). The full contents of the supplement are available online at http://www.biomedcentral.com/bmcmedgenomics/supplements/8/S2.
